# The impact of artificial intelligence on event experiences: a scenario technique approach

**DOI:** 10.1007/s12525-020-00433-4

**Published:** 2020-09-07

**Authors:** Barbara Neuhofer, Bianca Magnus, Krzysztof Celuch

**Affiliations:** 1 Innovation and Management in Tourism, Salzburg University of Applied Sciences, Campus Urstein Süd 1, A-5412 Puch/Salzburg, Austria; 2 Faculty of Economic Sciences and Management, Nicolaus Copernicus University, ul. Gagarina 13a, 87-100 Toruń, Poland

**Keywords:** Artificial intelligence, Customer experience, Service dominant logic, Value co-creation, Events industry, Scenario technique approach

## Abstract

Digital technologies are transforming human relations, interactions and experiences in the business landscape. Whilst a great potential of artificial intelligence (AI) in the service industries is predicted, the concrete influence of AI on customer experiences remains little understood. Drawing upon the service-dominant (SD) logic as a theoretical lens and a scenario technique approach, this study explores the impact of artificial intelligence as an operant resource on event experiences. The findings offer a conceptualisation of three distinct future scenarios for the year 2026 that map out a spectrum of experiences from value co-creation to value co-destruction of events. The paper makes a theoretical contribution in that it bridges marketing, technology and experience literature, and zooms in on AI as a non-human actor of future experience life ecosystems. A practical guideline for event planners is offered on how to implement AI across each touch point of the events ecosystem.

## Introduction

The advent of artificial intelligence (AI) and subsequent transformation of the global business landscape has been predicted for several decades, portraying it as one of the most disruptive technologies over the next 10 years (Panetta 2017). Despite the potential of artificial intelligence, several questions are raised: How will AI improve over the next years? Will AI be able to surpass human intelligence, and in which industries can it be applied? Today, we witness AI on the market in form of robots, virtual assistants and self-driving cars, permeating our everyday lives (Tegmark 2017; Murphy et al. 2017; Devlin 2018; Wirtz et al. 2018) and allowing businesses and customers to take advantage of the technology in its early stages (Sicular and Brant 2018; The Future of Life Institute 2018).

In contributing to a critical discourse around AI, researchers have questioned whether these latest technological advancements are creating real value, or whether AI may be overhyped. In this context, Ford (2018) coined the notion of ‘AI winter’, highlighting the existing discrepancy between market breakthrough predictions and actual progress. While the initial seeds of AI can be dated back to the early 1980s, it is evident that today AI has achieved progress across multiple industries with a potential to perform even better (Ford 2018). For instance, AI has been implemented in medicine (Becker 2019), manufacturing (Lee et al. 2018), the service fields and tourism context (Ivanov and Webster 2017; Huang and Rust 2018; Tussyadiah and Miller 2019).

Among the world’s industries, the service, tourism and events industries have always been at the forefront of digital advancement (Buhalis and Law 2008; Martin and Cazarré 2016). The proliferation of latest information and communication technologies (ICTs) has led to the rise of smart and intelligent solutions applied as resources in interconnected business environments and ecosystems (Gretzel 2011; Neuhofer et al. 2015; Gretzel et al. 2015; Femenia-Serra et al. 2018). The events industry has been pioneering in the arena of digital technologies. For instance, large-scale events have used various forms of ICTs, such as online ticketing solutions, event apps and wearable devices to engage customers (Solaris 2018) and to create outstanding customer experiences and value (Martin and Cazarré 2016; Backman 2018). In addition, we can witness sprinkles of avant-garde AI applications (e.g. event bots) across the industry. The future potential of events however depends on how quickly AI evolves. By envisioning events that are highly customised to user preferences, scholars suggest that event organisers will move away from having dedicated event apps to delivering content through messaging platforms via personal event bots (Davidson 2019).

While the major potential of AI is predicted across the service industries (Ivanov and Webster 2017; Huang and Rust 2018; Kaartemo and Helkkula 2018), its application remains however largely theorised and little understood in practice. Kaartemo and Helkkula (2018) offer one of the most comprehensive studies of AI in the fields of service science, business and marketing research. In their systematic review of the literature (see Kaartemo and Helkkula 2018), they set an agenda for AI research in value co-creation with the following key areas: 1) generic field advancement of technology in value co-creation, 2) AI and robots in a service provider’s value co-creation, 3) AI and robots in a beneficiary’s value co-creation, 4) AI and robots in systemic value co-creation, 5) shopping bots in value co-creation, 6) autonomous shopping devices (shopping bot 3.0) in value co-creation, and 7) post-phenomenological research on AI and robots in value co-creation.

In line with the identified gap of this study, Kaartemo and Helkkula (2018) argue that while technology is a key area in contemporary service-dominant (SD) logic studies, technology-mediated value co-creation is still often limited to a discussion of humans as actors. What we need are studies that transcend actor discussions toward human-to-non-human value co-creation (Gidhagen et al. 2017), and explore how AI could become a non-human actor with the potential to transform markets and ecosystems. Based on this gap, this study seeks to contribute to areas 1 and 2 (Kaartemo and Helkkula 2018) in that it aims to create a better understanding of how AI serves as a resource and potential non-human actor in human-dominated business context, i.e. events experiences.

Current technology literature suggests that due to the high amount of uncertainties in the future development of AI (Ford 2018; Sicular and Brant 2018), it is impossible to predict the exact manifestation of AI. What is however possible is to explore the status quo of AI and make informed predictions of possible future scenarios of AI in a business context. This study adopts a SD logic lens to zoom-in on AI as a non-human actor that may transform the future of event experiences. To uncover its potential implementation, a futures methodology through a scenario technique approach is used to map out three future scenarios of AI across all stages (pre, during, post) within the event experience journey and wider ecosystem. A practical guideline illustrates the main implications and provides an overview for event planners.

## Theoretical background

### Artificial intelligence

Most definitions tend to explain artificial intelligence in analogy to human intelligence. In the 1950s, the British computer scientist Alan Turing raised the question “Can machines think?” and therefore built a basis for the comparison of human brains and machines (Turing 1950; Mohammed et al. 2016). Describing AI as a science of creating intelligent machines (Nilsson 2010; Gretzel 2011) does implicate the term ‘intelligence’, which can be understood as the ability of solving complex tasks (Tegmark 2017), learning from action towards specific objectives, and functioning with foresight in an environment (Nilsson 2010; Gretzel 2011).

In an attempt to capture the wider impact of AI, society faces important socio-economic questions of whether and how machines can be intelligent, or we ought to redefine the way we think about intelligence. The intelligence of systems is usually judged against our understanding of human intelligence (Gretzel 2011), and distinguished as such, leading to a three-dimensional categorisation:

1. Narrow or weak AI (Kurzweil 2005) is designed to recognise faces, drive cars and provide assistance through chatbots, voice assistants and service robots, thereby performing specific tasks better than humans do (Murphy et al. 2017; Tegmark 2017; Van Doorn et al. 2017; Devlin 2018; Ivanov et al. 2019).
2. Artificial General Intelligence (AGI) is able to surpass humans at every cognitive level (Carrico 2018). AGI represents a machine that has the capability to generalise knowledge through different domains and to reflect on itself (Goertzel and Wang 2007). The gap between narrow AI and AGI becomes visible with the example of IBM’s Deep Blue System (Campbell et al. 2002). While Deep Blue defeated the world chess champion, Gary Kasparov, it did not manage to transfer these skills to other tasks without the need for human reprogramming. This implies that AGI improves itself to the limits of accessible data.
3. Artificial Superintelligence (ASI) does not know any limits and exceeds human brain capacity in every aspect (Kurzweil 2005; Tegmark 2017). Hitherto, AI has not reached human level yet.

Among these forms of AI, development stages and capacities differ greatly, with AGI currently being considered a theoretical future technology. Leading global experts, such as Demis Hassabis (Google DeepMind founder) and Jeff Dean (Leader of Google’s AI division) estimate to achieve a human-level machine (AGI) with a 50% chance by 2099 (Ford 2018). Despite these conservative predictions, Mark J. Walker (Research director of Gartner Inc.) identified AI as one of the megatrends and expects it to be “the most disruptive class of technologies over the next 10 years” (Panetta 2017). In fact, AI is already omnipresent across research and industries and has permeated our everyday life activities, while we may not always be aware of it (Tussyadiah and Miller 2019). For instance, voice-activated assistants, including Apple’s Siri, Google’s Allo, or Google’s Duplex are only some of the latest examples that demonstrate how AI already finds application in a wide range of situations to enable more personalised services on a daily basis (Tussyadiah and Miller 2019).

This makes it critical to understand how AI is transforming everyday life and consumption encounters. The service sector and tourism are increasingly reliant upon intelligent technological solutions that understand and react to human needs (Gretzel 2011). The reason behind the estimated high potential of AI in travel, tourism and events primarily lies in its ability to assist in recognising voices, faces and sounds, in facilitating tailored services, and in making predictions of future purchase actions.

With ever expanding abilities, AI has unprecedented possibilities to assist businesses, increase efficiency and reduce costs, while making human lives easier, enhance experiences and create added value (Gretzel et al. 2015; Tung and Au 2018; Tussyadiah et al. 2018; Tussyadiah and Miller 2019). These capabilities render AI a promising resource for experiences, especially when it matters to get to know customers, track user behaviours, use data in real-time, make suggestions and offer superior value propositions *in-context* and *in real-time* – all scenarios, which are particularly relevant for designing high quality events.

### Service-dominant logic and value co-creation

In following the footsteps of Vargo and Lusch (2017), a SD-logic perspective is adopted as the underpinning theoretical lens. The SD-logic is central to the contemporary marketing discourse, and offers a valuable approach to understanding the dynamics of actors and resources in experiences, value co-creation and interconnected service ecosystems (Wieland et al. 2012; Akaka and Vargo 2014; Vargo and Lusch 2017; Ramaswamy and Ozcan 2018). The core premise of the SD-logic is that firms do not merely deliver services, but instead offer value propositions and resources (e.g. skills, competences, technologies), which form the foundation for actors (Lusch and Nambisan 2015; Storbacka et al. 2016) to engage in mutual co-creation of value in-context and in-use (e.g. services, retail, events) (Wieland et al. 2012; Lemon and Verhoef 2016; Vargo and Lusch 2017). Value requires the integration of specific resources, conceptually distinguished between operant resources (knowledge and skills) and operand resources (materials), which are dynamically integrated for value to be successfully realised (Storbacka et al. 2016; Vargo and Lusch 2016).

With fast-paced developments at the technological frontier, the SD-logic is more relevant than ever. Experiences and value co-creation are technology-mediated on an unprecedented scale throughout the entire customer journey, before, during and after experiences, and simultaneously in the physical and digital sphere (Cabiddu et al. 2013; Neuhofer et al. 2015; Ramaswamy and Ozcan 2018).

This is where the SD-logic serves as a bridge to conceptually unite marketing and technology literature. Information technology has been discussed as a resource since the 1990s (Orlikowski 1992). However, only most recently, scholarship has started to open a fresher and more topical debate on the nature of technology as a resource in service systems, value co-creation propositions and innovation processes (Akaka and Vargo 2014; Lusch and Nambisan 2015; Ramaswamy and Ozcan 2018). In this vein, Akaka and Vargo (2014, p.368) define technology as “a collection of practices and processes, as well as symbols that are drawn upon to serve a human purpose”. Tourism destinations, hotels and events represent only some of the contexts in which digital technologies have become integral to experience and value propositions along the entire customer journey (Neuhofer et al. 2015; Martin and Cazarré 2016; Tussyadiah et al. 2018).

In order for value realisation to happen, all resources need to be properly accessed and integrated, whether it is skills or technology. For many years, the service marketing literature focused on the integration of resources towards the co-creation of positive value. This discussion however missed one important component, namely the possibility that something at some point goes wrong. Plé and Chumpitaz Cáceres (2010) were among the first to point out possible negative outcomes of resource integration, suggesting that value may not always be co-created, but may sometimes be co-destroyed. In fact, it is unrealistic to expect the interaction of actors and application of resources to be merely positive. This prompted scholars to call for a more nuanced understanding of value formation, extending the spectrum from value co-creation (positive), towards no-creation (neutral), and to value co-destruction (negative) (Marcos-Cuevas et al. 2014; Neuhofer 2016; Makkonen and Olkkonen 2017; Camilleri and Neuhofer 2017; Järvi et al. 2018).

It is the incongruence between actors, their practices and resources that could destroy an experience, whether intentionally or involuntarily (Plé and Chumpitaz Cáceres 2010; Echeverri and Skålén 2011). This scenario is also true for the integration of advanced technologies resources (e.g. AI), which may lead to value co-creation or co-destruction when a system is not ready, well developed or working properly (Neuhofer 2016). The events industry has always been a playground for innovations in experiences and technology, and has recently seen a surge of cutting-edge technologies put into place (Cooper 2018; Global Event Technologies 2019).

### Connecting the dots: AI, event experiences and value co-creation

The events industry is known to dynamically embrace changes in the environment (Bowdin et al. 2012; Getz 2012; Robertson et al. 2015), to meet and succeed attendees’ expectations, and to deliver outstanding experiences (Pine and Gilmore 1999). One such area of change represents the proliferation and implementation of state of the art technologies, introducing a new era of event technologies (Solaris 2018). In taking a look at the evolution of event technologies, Solaris (2018) categorises four waves of development: (1) online registration and ticketing, (2) event mobile apps, and (3) engagement technology (polls, apps, live engagement). The first three waves already reached the status of experience commodity (Pine and Gilmore 1999), and represent a “norm”, whereas the fourth creates a new ecosystem by adding (4) Virtual Reality (VR), Augmented Reality (AR) and Artificial Intelligence (AI) (Solaris 2018).

These new technological players are predicted to not only revitalise and change some aspects of the previous waves, but also create entirely new possibilities for event experience design (Solaris 2018). Latest research underlines the central role that technologies (e.g. augmented reality, wearables, smart systems, AI and robots) play in facilitating contemporary experience and value propositions (Tung and Au 2018; Tussyadiah et al. 2018; Ivanov et al. 2019). In fact, the experience which event attendees demand from events has changed (Robertson et al. 2015; Martin and Cazarré 2016), with individual actors expecting to use technology to support, co-create and personalise their experiences (Neuhofer et al. 2015; Lemon and Verhoef 2016). The event context is rich in examples that show how event planners use smart access, payment systems, and event apps to enhance the attendees’ experiences and keep them up-to-date (Global Event Technologies 2019; LineUpr 2019).

Connecting the dots towards the aim of the study, namely understanding the impact of AI on event experiences, poses the question which new experiences we may witness through the application of AI (Cooper 2018). Research into AI in event ecosystems is scarce to date, with most research epitomising a broad scope of technology in generic (tourism) service encounters (Huang and Rust 2018; Kaartemo and Helkkula 2018; Tussyadiah and Miller 2019). For the events industry, knowledge around AI at events is mostly found in the realm of event experts offering opinions, predictions and trend reports online (e.g. Groot 2017; McCorkell 2017; Cooper 2018). A synthesis of their reports shows that AI is already being used in form of chatbots, event apps, predictions, virtual concierges (suggestions, reminders), instant translation apps and personalisation. These industry examples display the great potential of AI in the future, and in turn, underline the need for its investigation (Van Doorn et al. 2017; Huang and Rust 2018; Tussyadiah and Miller 2019).

## Research design

A scenario technique approach was adopted to identify the future of AI and how its application may lead to potential value formation in event experiences. A futures methodology is particularly valuable because it relates to developments in the long term. It enables to expand and order the perceived range of possibilities by constructing a series of scenarios developed through the comparison of trends and uncertainties (Schoemaker 1995; Yeoman et al. 2011). The goal of scenario planning is to predict multiple uncertain future scenarios (Van der Heijden et al. 2002), which differ fundamentally from each other, while they do not cover all possibilities exhaustively. Instead, they circumscribe possible situations and provide a simplification of these outcomes (Schoemaker 1995).

First used in the 1960s by Hermann Kahn as a tool for business prognosis, its applicability is proclaimed to “virtually any situation in which a decision maker would like to imagine how the future might unfold” (Schoemaker 1995, p. 27). In the marketing field, futures methodologies have been recommended and adopted for strategic decision-making and leadership (Lew et al. 2019), for innovation studies (Orazi and Cruz 2018), for tourism sustainable planning (Yeoman et al. 2015), and most recently for mapping COVID-19 related risk and crisis management (Cankurtaran and Beverland 2020). Within an electronic marketing context, Rincon et al. (2017) propose the scenario technique as a particularly valuable, yet sparingly adopted, method when it comes to understanding emerging technologies. In their study, they used a scenario technique approach to explore the future of wearable devices in tourism and developed four possible scenarios showing how the tourist experience might be affected.

In this study, qualitative data was gathered through six interactive focus groups. A purposive sampling approach was adopted to recruit participants based on the criteria of individuals a) basic knowledge of AI and b) working in technology-intensive or events-related firms. Thereby, participants were asked to conduct a self-assessment of ‘technological understanding’ from 1 to 5. A total of 33 participants were recruited, representing a broad range of expertise, including potential event attendees, AI experts, event practitioners as well as service and event management academics. Table 1 provides a socio-demographic overview of the study’s participants.

**Table 1:** Socio-demographic overview

Variables	Potential event attendee	AI expert	Event practitioner	Academic service/Event management	Total
Participants (n=)	13	3	8	9	33
Age (22–44, mean)	28	31	30	27	29
Gender (%)					
Male	15.4	100.0	75.0	55.6	45.4
Female	84.6	0.0	25.0	44.4	54.6
Level of education (%)					
High School	53.8	33.3	25.0		30.3
College/University	46.2	33.3	50.0	88.9	57.6
Master’s or PhD		33.3	25.0	11.1	12.1
Technological understanding (mean)					
1–5 (Excellent to no understanding)	3.4	1.3	1.6	2.6	2.6

The focus groups, conducted in March 2019, encompassed five to six participants in each group and lasted between 70 and 90 min. Each workshop was documented through voice recording and written flipcharts. A workshop protocol was developed that followed the structure: (1) explanation of the research, underpinned with an introduction to AI, (2) warm-up discussion about the participants’ experience with technologies in the events industry, (3) independent brainstorming activities about the implementation of AI along the customer journey (pre, during and post event), followed by (4) a discussion about the driving forces and concerns of the experience.

### Scenario data collection and analysis process

The scenario development process followed Fink and Schlake (2000), who suggest five phases, including (1) Scenario Preparation, (2) Scenario Field Analysis, (3) Scenario Prognostics, (4) Scenario Development and (5) Scenario Transfer.

#### Phase 1: Scenario preparation

The first step of the scenario approach was to build a *scenario base* (Fink and Schlake 2000), a time frame and a scope of the analysis (Schoemaker 1995; Godet 2006). Due to the study’s focus on supporting decisions in a business environment, the *decision field* of AI along the event experience (pre, during, post) builds the core of the scenario management process. The position of AI technologies on the 2018 Gartner Hype Cycle for Emerging Technologies (Sicular and Brant 2018) assumes AI to reach the plateau of productivity in five to ten years, which determined the future scenario horizon of seven years (2019–2026).

#### Phase 2: Scenario field analysis

A clear demarcation of the *scenario field* adds precision to the prior developed decision field. The outcome of the second phase was a total of 287 distinct factors that emerged of the structural analysis (six focus groups), which aimed to show the influence of AI as a resource in event experiences. Each workshop followed the same structure, as explained in the protocol above.

The developed 287 factors (e.g. “aspire attention”, “marketing”, “parking assistant” and “automatic reservation”) were further summarised in a cluster analysis through which similar factors were grouped together to 23 variables (e.g. “Type of Technology”, “Booking/Registration”, “Information” and “Self-Driving Transport”) representing different forms of the same concept (Van der Heijden et al. 2002). The clustering followed the structural analysis method, which seeks to highlight the key variables influencing the field of study with the help of a cross-impact matrix (Godet and Meunier 1999).

According to that, the relationships between the variables were rated pairwise on a scale from 0 (no influence) to 3 (strong influence) (Schüll and Schröter 2013), which resulted in ten distinct key drivers. To validate this outcome, four selected experts from the field of events and technology at events were invited to perform an independent cross-impact matrix analysis to extract the average factor for the subsequent ranking. The determined key drivers were: *(1) Suggestions/Assistance, (2) Tracking of Customer Behaviour, (3) Type of Technology, (4) Event Organisation, (5) Personalised Experience/Individualization, (6) Crowd Management, (7) Failure of System, (8) Orientation/Smart Guidance, (9) Security Concept/Surveillance and (10) Marketing/Targeting.*

#### Phase 3: Scenario prognostics

Following the selection and ranking of ten key drivers, step three contained a first look into the future within the scenario process. A morphological analysis was conducted. For this purpose, two polar outcomes (value co-creation and value co-destruction) were identified for each key driver (see Table 2 below and Table 3 in the Appendix), supported by a description based on participant statements (Van der Heijden et al. 2002). Fink and Siebe’s (2016) guidelines were followed to build first scenarios and future projections for each factor. Key driver (1) Suggestions/Assistance is exemplified below, showing a polar projection and a description.

**Table 2:** Example of polar spectrum of factor 1

Example factor 1: Suggestions and Assistance	
Polar spectrum	Description
Projection A: Value co-creation (positive)	Attendees receive individual suggestions and assistance according to their needs. They can save time and make better decisions. AI improves the whole process and enhances the experience.
Projection B: Value co-destruction (negative)	Visitors get constant suggestions, which do not fit their needs. An overload of information leads people to become annoyed and feeling as though their experience and value is being co-destroyed. Participants miss some of their preferences, because the system gave wrong information.

#### Phase 4: Scenario development

**Fig. 1:**
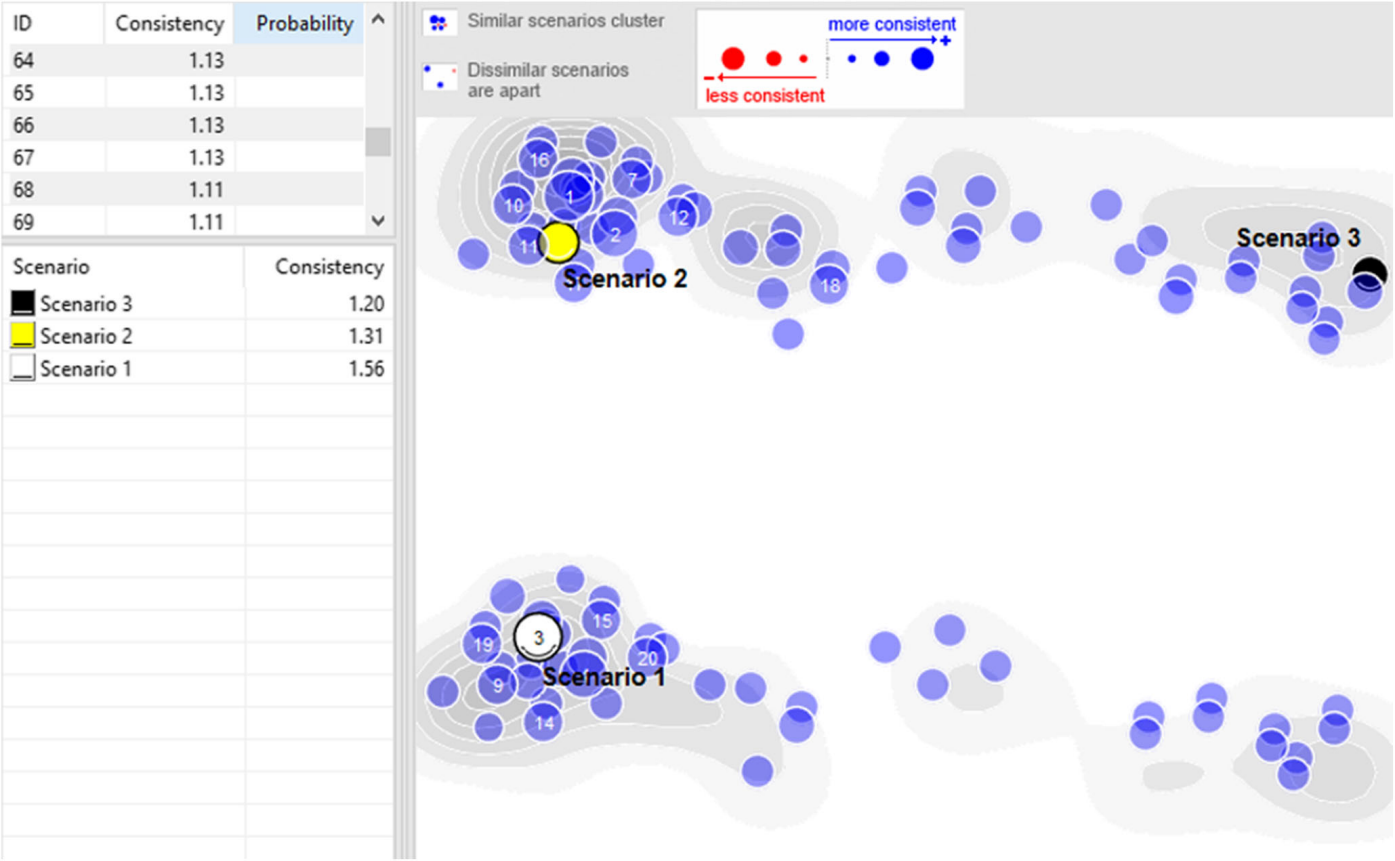
Scenario cluster visualisation with Parmenides Eidos software

In order to test the plausibility and consistency of the scenarios, all projections were rated pairwise on a scale from −3 (=completely inconsistent) and + 3 (=consistent with effect enhancement) (Schüll and Schröter 2013; Fink and Siebe 2016). Following the manual morphological analysis, a computer-assisted scenario planning was conducted with the software Parmenides eidos, which served as a tool to calculate all different possible combinations. Parmenides eidos calculated over 1,000 different scenarios. The first hundred scenarios were explored further. To further reduce the amount of scenarios, Van der Heijden et al. (2002) emphasise the fundamental importance of the scenario’s plausibility and consistency. Scenarios where chosen according to a high factor of consistency, and to represent a maximum variation of different futures rather than variations of the same (Schoemaker 1995; Schüll and Schröter 2013). In their respective studies, Yeoman et al. (2015) and Rincon et al. (2017) defined four distinct scenarios, while Schoemaker (1995) suggests that the final number of chosen scenarios is mostly based on the quality of the outcomes. Parmenides eidos was used to visualise the most contrasting scenarios, furthest apart, with the highest rate of consistency, which resulted in three strong distinct scenarios (see Fig. 1).

#### Phase 5: Scenario transfer

The final step of the analysis includes the scenario transfer, which serves as a strategic tool by looking at the scenarios’ impact on the determined decision field (Fink and Schlake 2000). The decision field was the business environment AI in the events industry. This phase generated three distinct scenarios, which were subsequently developed into plausible stories.

## Results

**Fig. 2:**
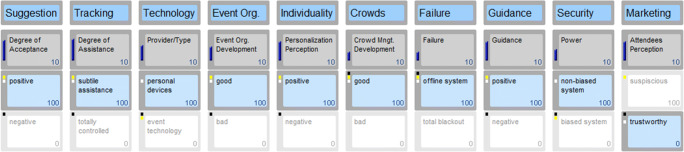
Scenario 1: A personalised event experience

**Fig. 3:**
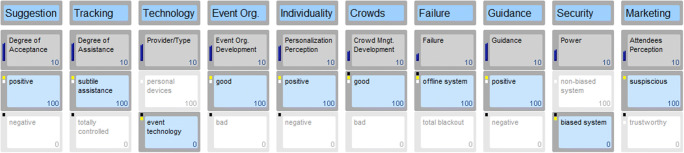
Scenario 2: A suspicious event experience

**Fig. 4:**
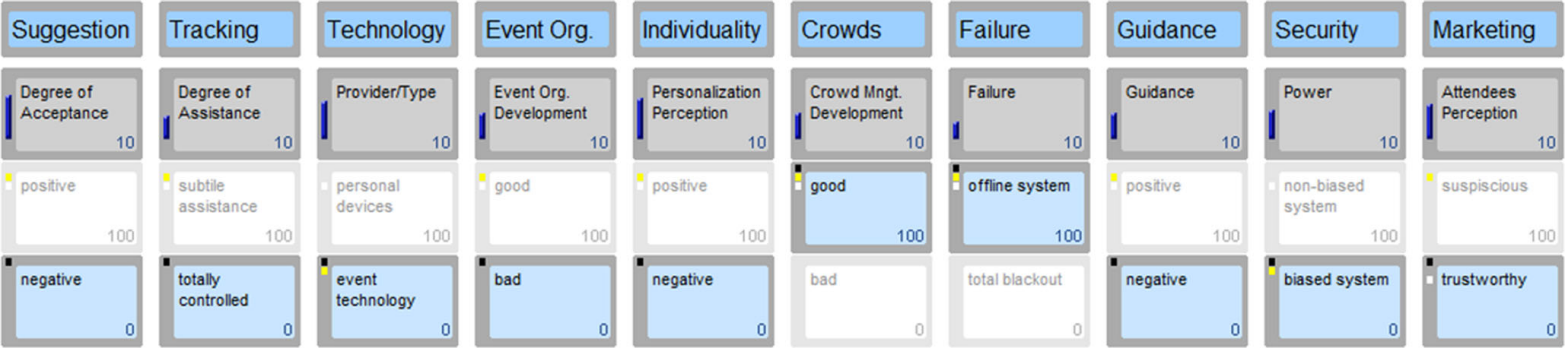
Scenario 3: Some badly developed systems

The final three scenarios were named after their contrarian approaches, as 1) ‘*A Personalised Event Experience’* (value co-created), 2) *‘A Suspicious Event experience’* (value no-created/co-destructed) and 3) *‘Some Badly Developed Systems’* (value co-destructed). The presented scenarios were created based on a maximum variation of the key drivers, and are visualised in Figs. 2, 3 and 4. Following Yeoman et al.’s (2015) approach, the concept of storytelling was employed as a means to add personalisation and open the mindset for a stimulating discussion and further thoughts rather than a tunnel viewed future. All mentioned characters and events are fictional, and stories take place in a frame of seven years from the time of the study, in the year 2026. Comments written in italics represent participant quotes, which have been embellished in their form, without changing their meaning.

### The event dot.com

In order to illustrate a realistic future scenario, the researchers created a fictional event called *“dot.com”.* Dot.com represents an international event for visionaries, game changers and people interested in the future. The event takes place on April 27th, 2026 in Vienna. In a frame of 12 h, event attendees are able to listen to keynote speeches, visit stands of innovative start-ups, expand their network and enjoy several music acts. Dot.com is a hub for future-oriented digital projects and a stage for emerging start-ups. Participants of all ages, industries and communities network and share their knowledge. Different areas allow moving around freely and joining any type presentation or act. To provide an excellent experience and value, the event organisers implemented several touch points through state-of-the-art technologies, predominantly AI. For instance, access areas use face recognition, bar staff is replaced by robots and participants can connect their wearable devices and technologies with the event system in place. The scenarios cover the entire pre, during and post event experience. Three scenarios follow next, with Figs. 2, 3 and 4 highlighting a variation of the ten key drivers determining each distinct scenario.

#### Scenario 1: A personalised event experience

Eva, a 31-year-old German founder of an online start-up, is surfing through social media and listening to her favourite playlist when suddenly Nelly, her virtual assistant, appears on the screen. The virtual assistant is constantly helping Eva in case she needs any information, translation, phone calls or tasks accomplished. Nelly basically “*guides her through life”*. Nelly suggests Eva the upcoming festival *dot.com* in Vienna, where she is able to see visionary keynote speakers, interesting potential business partners and some famous acts relating to her fields of interest. She also mentions that the early bird registration phase is over soon and Eva should decide whether she wants to attend and save some money. Eva feels very positive about the suggestion, since the event fits her needs perfectly. Nevertheless, to strengthen her decision to go, she puts on her VR-glasses to check the atmosphere at the venue. After re-living the event of the previous year, she decides to attend. A simple “yes, I would like to attend” makes Nelly carry out the task and she proposes Eva a ticket category tailored to her needs. Eva places the tip of her right index finger on the laptop and the transaction is accomplished. Implanted microchips have been a “thing” in the last two years. All the scepticism about *“transparency, security and data protection”* of society vanished as soon as it became mainstream.

A few days before dot.com, Nelly assists Eva to plan the trip to the event. Simple yes/no-questions make Nelly learn how Eva wants to experience the event. On the way to the venue, the assistant suggests a little coffee break. The measurement of Eva’s health data shows that she should have a bit of caffeine and in addition, the parking spaces and entries at the venue are very crowded at the moment. Once arrived at the site, Eva has a smooth access experience when *“the system automatically recognised her”* and access was granted within seconds. At the event venue, Eva is free to use her own devices, such as microchips, smartphone and wearables, depending on her preferences. Once through the access gates, Eva tries to get an overview of the area. Before she gets lost, a push notification on her phone *“recommends a tailored schedule”*, based on keynotes, contacts and acts, which appeal to her most currently, along with new suggestions, which she may profit from in the future. Soon, Nelly points out that Eva should better get some beverages to stay hydrated during the presentation and then immediately go to the first keynote, since a large crowd is already intending to move towards the same room. During the speech, Nelly notices Eva’s enthusiasm and encouragement for the speakers and directs additional information about the subject directly to her mail inbox. By doing so, Nelly *“eliminates the need for brochures and other printed materials”*, which Eva would have to physically carry around at the event.

During a break, Eva is curious whether there are some friends or acquaintances attending the event to meet for a chat. Eva uses her microchip again and puts her finger on the *“social board, where you can find your social groups”*. The social board is located in the centre of the venue and allows attendees to track related people. Successfully done, Eva meets a former colleague and they decide to go for a drink. At the bar *“which is without staff and handled by the attendee”*, Eva receives a drink with a customised amount of alcohol, still allowing her to drive her car, but enough to rise her mood and have a good experience. Payment takes place through the microchip and is automatically deducted from her bank account. Some hours later, Eva receives another push notification with special offers of merchandise products and a hint, which stands she has not visited yet. Eva feels in good hands and thinks: *“wow, the thing knows what you want”* and follows the call to the merchandise. Since Nelly knows her size and look, Eva *“does not have to think of: what fits myself, which size should I choose. Instead, it shows you immediately how it fits you”.*

Back home, Eva receives a complete digital customised package with a range of info material and personalised media (e.g. pictures, after movie) plus drafts of recommended posts for her social media channels. In addition to this created value, Eva can take a look at the health and transactional statistics of her implanted microchip. She knows exactly about her nutritional consumption, how many steps she walked and how her body reacted to keynotes, acts and social interactions. A review of dot.com uploads automatically, based on that data. It is not necessary to read or write any reviews, as Nelly already incorporates these into her decisions and suggestions. Thinking back to the event a few days later, Nelly pops up again with a suggestion for a friend request with a person Eva met at the event, who has a 98%-matching rate. Eva smiles. What a personalised event experience.

#### Scenario 2: A suspicious event experience

Eva is slowly using her virtual assistant Nelly more and more. Even though it works perfectly, Eva has problems trusting her device. She knows that she reveals a lot of data due to her media usage, which is why she feels observed when using active assistance and sometimes *“gets terrified when she recognises how transparent she is”*. Just recently, Nelly has proposed the event dot.com, which is perfectly attuned to Eva’s needs and she has decided to attend. After a successful ticket purchase, which she made on her smartphone, she is now preparing for the trip. By purchasing the ticket, Eva was sent a wristband, which grants her access to the area, allows payment and supports any infrastructure offered at the event location. Eva can also connect the wristband to her smartphone, so she can keep track of her activities at any time. To get to the location, Nelly proposes taking the shuttle bus, which is very expensive in comparison to other public transport. Several times, Eva tries to receive a better suggestion for transport, but Nelly insists the shuttle to be the best solution. *“What is going on? It is my decision, not yours!”*, Eva swears. In the end, she follows Nelly’s recommendation.

After arriving at the event site, Eva is granted access by a face recognition system. *“All the data is recorded. Who is going to use it and in which way? They could use it for their benefit and if the system has bad intentions, it could misuse all the data to create fake news about me…”.* With those thoughts in her mind, Eva is confronted with the next technology: security robots who monitor the entrance and *“automatically check the bag on illegal items”.* Eva simply cannot make friends with service robots, although they have already been in use at most events since 2024. She thinks they are *“scary”*. After the fast-processed access, Eva first wants to find her bearings and uses the help of a service robot. This explains exactly which keynote takes place where and how to get there. The robot transmits all necessary information to Eva’s smartphone, so she is not bound to the robot’s location, but can follow the instructions on her smartphone independently. After a while of following the advice of Nelly, Eva has already spent a lot of money. All suggestions seem to make sense and appeal to her, so the experience and value created outweighs her consciousness. On her way to the food and beverage area, Eva comes to an info-point, where she is able to check-in with her wristband and *“receives information about the weather, acts, infrastructure, personal data, consumption”* and so on. Eva recognises a section where she gets *“special offers and vouchers customised to her needs”.* Most of the offers increase her willingness to purchase more and send her to areas, where she has to pay additional entry fees, resulting in higher expenses. She feels a little bit suspicious regarding the marketing techniques, but still decides to enjoy the event and consume according to her personal preferences.

Two hours before the event ends, the alarm goes off and the attendees are asked to leave a certain area and follow the instructions of the security robots. The uncertainty of the visitors is noticeable and expressed by extreme tension. Everyone is focused on their smartphone and tries to get information about a possible incident. The security robots are giving more and more instructions and people are unsure whether they should follow them, but also do not dare to contradict. Eva has heard several stories of robots *“spreading fake news to force people to certain actions”* and is again suspicious. An accident team is able to get to the emergency just in time and the security robots assist in clearing the way. By *“tracking health data”*, the control centre was able to detect the collapse of an event attendee early and intervene accordingly.

Although the situation was largely positive, Eva’s suspiciousness increased even more because she feels as though the device controls her and helps boost the profit of the organisers, rather than creating value for herself. Back home, Eva immediately checks her historical data of the event and is surprised by how many tailored offers she got that fit perfectly to her needs. All in all, she remains suspicious. She is not sure whether all offers were based on the intention of offering her the “perfect experience”, or whether they are just about increasing revenue for the event organisers.

#### Scenario 3: Some badly developed systems

Event bots, virtual assistants and event apps have reached acceptance in society and in the events industry. Eva is familiar with these types of technologies, nevertheless, does not use them very often. This might change because of an upcoming event. Eva’s friend Ben is into every kind of new technology and names himself an early adopter. He invited Eva to come along to an event called *dot.com*, where those technologies are already being used. The invitation arrives via Nelly, a rarely used virtual assistant on Eva’s smartphone. Eva gives it a try and accepts the invitation. A short scan of her face, conducted with the front camera of her phone, promises to grant her access when arriving at the event location *“without even showing your ticket”*. Since Eva has no idea what to expect at the venue, she gives the browser-based chatbot a try. After several confusing answers, which led back to the homepage of the event website, she gives up.

Eva arrives a few minutes earlier than Ben and uses the time to get familiar with the technology. Signs at the entrance advise the event attendees to log into the event system with their own devices in order to create the perfect experience. Eva follows the recommendation and is soon connected to the system. Immediately, a first push notification pops up and tells her to access the venue soon in order to avoid missing some keynote speakers. The event has not started yet. So Eva wonders why the system already puts pressure on her and *“tries to guide her in a certain direction”*. Finally Ben arrives and they aim to enter the event area. The *“access is handled totally staff-free”* through robots and face recognition, which immediately leads to difficulties. The system does not recognise Eva’s face, although she registered via her smartphone. Several attempts fail. Eva tries to find a real person and after 10 min of trying, a staff member shows up, who is not able to help, since he is overwhelmed with the technology. The staff member grants them access via another gate and tries to smooth the mood of Eva and Ben by giving them free drinks and crediting them through the event system.

Eva connected her personal devices to the event system. Now she is constantly receiving push notifications and suggestions from Nelly. The system tracks her every step and depending on where she is, she gets a call to action. Annoyed by the technology, Eva tries to find her favourite keynote speaker, which should speak in a few moments somewhere. Due to a lack of orientation at the venue, she tries to ask Nelly. The virtual assistant mentions some crowd issues and guides Eva to the presentation where she waits excited for the beginning. A few minutes after the start of the keynote, Eva wonders whether she is in the right room because the keynote speech is about a totally different topic. Soon she finds out that Nelly *“sent her to another room, according to a minor preference”*, because the first speech was too crowded. Eva missed her preferred speech. While leaving the room and searching for her friend Ben, Eva receives another push notification, suggesting her to visit the stands to collect information regarding her fields of interest. Once she makes a wrong turn, her device immediately warns her again and tells her what to do. *“This is too much for me, I feel overwhelmed”*. Eva “*feels totally restricted in her free will”* and decides to look for a place to get a drink and sit down. Finding her way to a bar, she orders a Gin and Tonic to soothe her mood. The bartender robot refuses to sell her a drink, justifying it to the fact that she is under 18. “There has to be an error in the system”, Eva responds, “I am 28.” Without any sign of empathy, the robot turns to another customer, leaving Eva standing at the bar. Eva waits for Nelly, to help her out, but of course – *“disappointment”*. Five hours before the actual end of the event, Eva decides to leave, since the *“technology destroyed her experience”*.

## Discussion of the results and outlook into the future

In order to illustrate the theoretical significance of the three scenarios from a positive to a suspicious to a negative experience, the approach of Yeoman et al. (2015) in “2050: New Zealand’s sustainable future” is adopted, which suggests to pose some significant ‘*So What*’ questions. The questions were developed based on the research questions, the literature and the data to a) make sense of the scenarios and b) contextualise and theoretically embed them into the wider AI and SD-logic value co-creation discourse (Gidhagen et al. 2017; Vargo and Lusch 2017; Huang and Rust 2018; Kaartemo and Helkkula 2018; Ivanov et al. 2019) towards a theoretical and practical contribution.

### How will AI shape the future of events?

The implementation of AI will have a major impact on the nature of experiences and value formation at events. Transcending the ‘enabling’ capacity of current information and communication technologies (Buhalis et al. 2019), AI serves as a game changer in that it becomes a key autonomous resource creating a new level of human-to-non-human interaction (Gidhagen et al. 2017). The scenarios show that attendees will have experiences characterised by a high level of personalisation through constant assistance. Especially the first scenario “A Personalised Event Experience” underlines Martin and Cazarré’s (2016) argument that the development of technology does imply a cyclical process, where everyday life and event experience melt together. AI is an all-encompassing non-human actor reaching into multiple life domains, as personal data and behaviour are tracked in everyday life and used in specific business transaction when a need arises. This would suggest indeed a transformation of current digital markets - one where business and life domains are no longer separated as AI facilitates interactions that permeate the boundaries of distinct domains. As a result, we propose that we will transcend insular service ecosystems (Wieland et al. 2012; Kaartemo and Helkkula 2018) and move towards more integrated life systems. We propose the novel term ‘*Technology-mediated Life Ecosystems’*. This human-centric ecosystem recognises AI-supported life realities of the end-user that cross and permeate all life domains. For events business ecosystems, this means that the customer journey and value chain shifts into the life domains of potential customers. With AI in place, a first business-customer (B2C) touch-point could thus be a push notification long time before the actual event that sparks initial attention and interest. Moreover, customised travel packages, individual transport offers and dynamic pricing will enhance the time prior the event. Since technology has found its way into the event industry through smartphones, access systems and apps (Solaris 2018), AI brings more agency as an actor (Kaartemo and Helkkula 2018), not only by facilitating but by shaping an experience and connecting all dots in one overall system. In the short-term, AI will mainly support the event experience by providing personalised recommendations, assistance and suggestions, and enhance event organisation in terms of logistics, crowd management and access systems. Long-term, the scenarios lead to suggest that AI redefines the business environment by substituting human-to-human actor interactions and by actively co-creating experiences for and with a customer.

### What are the main factors influencing the customer experience?

Scenarios one and two depict the significance of a *well-developed system* of AI in service contexts, drawing attention to potential value co-creation and value co-destruction outcomes (Makkonen and Olkkonen 2017; Järvi et al. 2018). The scenarios show that attendees will be annoyed if their high expectations are disenchanted when a system is not working properly. This is particularly important for the first-time adoption of and guidance by AI of an event experience. No matter which type of technology is provided, it has to be *accessible* with the *lowest effort possible*. The quality of the provided system goes hand in hand with the attendees’ *acceptance of technologies* (Davis 1986). People will be suspicious at first, nevertheless, the more they trust, the more they accept. Since event attendees use their private devices regularly, a certain level of trust lies in them. Providing or connecting event technologies via personal devices can therefore limit initial suspicion. This study found that the key difference between value co-creation and value co-destruction (Makkonen and Olkkonen 2017) is oftentimes the ‘*degree of interference’*. When acting as an agent and co-creator of an experience, AI needs to be carefully calibrated as to how much a system interferes in the attendee’s experience, with a fine line between overbearing intrusiveness and encouraging assistance. The degree of interference could thus be a novel component unique to observe and study in human-to-non-human actor value co-creation processes.

### Can AI co-destroy the customer experience and value?

As on operant resource and non-human actor (Akaka and Vargo 2014; Gidhagen et al. 2017), this study found that AI has the ability to potentially co-destroy value in several ways. While several recent SD-logic studies suggest value co-destruction (Makkonen and Olkkonen 2017; Järvi et al. 2018), this study finds nuances from complete value co-destruction to value co-reduction (Camilleri and Neuhofer 2017), as well as what we could term as ‘*ambivalent value co-creation’*, as AI is being perceived as suspicious and thus cautiously integrated with mixed value effects. For instance, scenarios two and three show that when AI solutions have malfunctions, they can diminish the customer experience. This may slow down processes, increase waiting times, create crowds and cause negative feelings (e.g. suspicion) among attendees. A further major negative outcome is ‘human social isolation’. Scenario one depicts how engaged the person is through use of AI. Acting as a non-human agent, AI makes decisions independently from other individuals since the human attendees receive suggestions tailored to their highly individualised needs and preferences, but different to others. The findings indicate that this might separate social groups and increase *isolated experiences* that diminish the social value of gatherings, personal relations and human experiences (Rihova et al. 2018). Furthermore, scenario three displays a risk towards the acceptance of technology (Davis 1986), namely the *fear of technology*. Especially, robots in service environments pose challenges for human-robot interaction among teams and customers (Murphy et al. 2017; Wirtz et al. 2018; Ivanov et al. 2019). Customers could be anxious towards robots, especially when used for security and safety management. Finally, event organisers need to account for potential *failures of the system*. No matter how far the technology has progressed and become intelligent today (Gretzel 2011), Yoshua Bengio, AI expert, confirms the need for a ‘*human in the loop’* (Ford 2018), who can intervene and assist in case of emergency. With increasing reliance on AI as actors, these steps will be critical to not only diminish the risks of value co-destruction but to avoid a service collapse of a fully technology-mediated (and reliant) business environment. Finally, AI does not (yet) have a moral understanding of what is right or wrong, which renders humans critical to keep an eye on all actions.

### How can event organisers implement AI as a valuable resource?

**Fig. 5:**
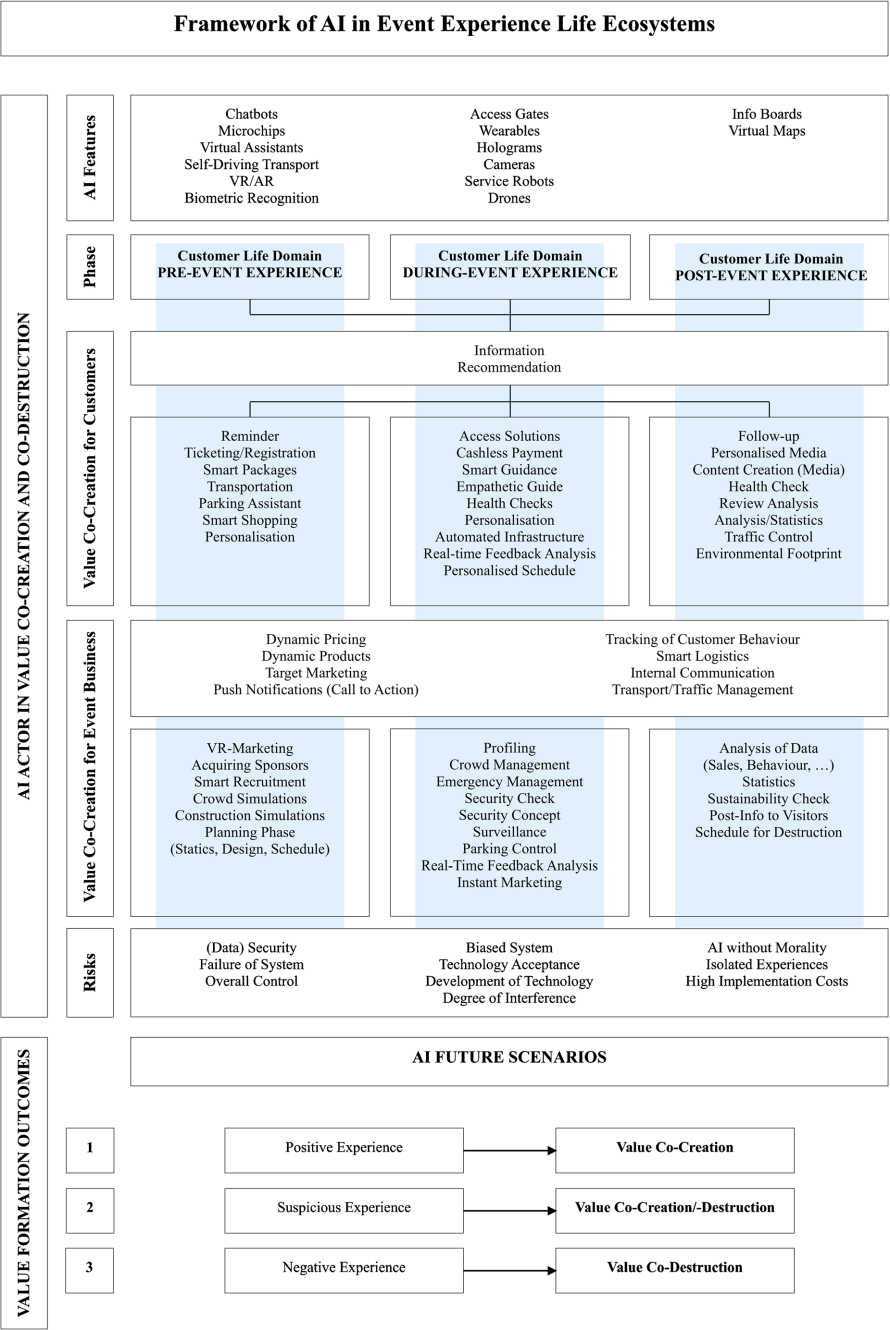
Framework of AI in the event experience life ecosystems

From a practical side, AI does raise opportunities and challenges for the events industry. AI is particularly valuable for *targeted marketing*, *customised packages* and *dynamic pricing*. Using behavioural data and tracking attendees’ actions does increase the chance to increase profits, while ethical considerations around data usage towards mutual value co-creation for businesses and customers will be critical. Scenario two highlights that *too many marketing actions weaken attendees’ trust*. Thus, every action has to transfer the feeling of creating value for the customer. For instance, AI could co-create value for customers by recommending ways to *save money* and *enhance experience flows*, while realising value for event organisers by creating *faster processes and cost savings*. A final consideration to address is *biased data* or *misuse of data*. Tracking attendees’ every steps comes with responsibility and requires a secure environment and reassurance that data are handled responsibly and to the value of the customer. Based on the three scenarios and foregone discussion, a model named ‘Framework of AI in Event Experience Life Ecosystems’ has been developed (Fig. 5). The framework maps out in detail AI as an actor of the event ecosystem, by depicting 1) its technological features, 2) the phase of the customer life domain pertaining to the consumption experience, 3) value co-creation for customers, 4) value co-creation for businesses, 5) value co-destruction risks, and 6) value co-creation/co-destruction outcomes of AI leading to a *positive*, *suspicious* or *negative* experience. By encompassing all stages of the customer experience (pre, during, post), the model holistically visualises the integration of AI as an actor that goes beyond the immediate transactions in B2C relations towards the life ecosystem of the customer. This insight opens a systems-focused discussion of service ecosystems (Wieland et al. 2016; Kaartemo and Helkkula 2018) and encourages a discussion on the symbiosis between human and non-human actors in service and value co-creation.

## Conclusions and implications

Artificial intelligence found its way into society and is predicted to become one of the most disruptive technologies over the next decade (Panetta 2017; Kaartemo and Helkkula 2018; Ivanov et al. 2019). Especially the service industries can benefit from latest technological developments, such as AI, by increasing productivity, supporting interactions and enhancing experiences (Van Doorn et al. 2017; Huang and Rust 2018; Ramaswamy and Ozcan 2018; Tussyadiah et al. 2018). The events industry is expected to be one of the business environments in which AI has great potential and we already witness ‘sprinkles of AI innovation’ on the global landscape.

### Theoretical implications

There is no question that AI brings disruption and transformation (Ivanov and Webster 2017; Buhalis et al. 2019). From a theoretical point of view, this study aimed to offer a glimpse into the future, and address the question of *how* does AI, as a non-human actor, transform service environments, i.e. events experiences, from a wider SD-logic and ecosystems perspective. Contemporary service science and marketing research is particularly concerned with understanding the ‘how’ behind value formation processes of novel technologies (Akaka and Vargo 2014; Vargo and Lusch 2016; Kaartemo and Helkkula 2018). This study mapped out the impact of AI on the events context as a business environment that is highly digitally enabled. The findings suggest that AI transcends the role of current ICTs (Buhalis et al. 2019) as a tool and resource that is merely *enabling*. In fact, AI transcends traditional technological capabilities and becomes an autonomous actor in experience and value co-creation together with its human counterpart. For business contexts, this suggests that AI transforms the fabric of current relations in that it re-shapes and substitutes touchpoints traditionally found in business-to-customer (Vargo and Lusch 2016) and customer-to-customer interactions (Rihova et al. 2018) and value co-creation processes (Kaartemo and Helkkula 2018). The future scenarios of the year 2026 indicate that AI takes precedence as an actor that becomes a primary experience facilitator and creator. For instance, AI actively proposes events, makes autonomous bookings, conducts purchases and determines the events itinerary and its touchpoints. As a result, AI has a transformational power that a) re-defines traditional actor interactions, b) shapes and influences human-to-non-human co-creation processes and c) extends service touchpoints beyond the immediate service ecosystem. Considering the omnipresence of AI, accessible through its various features and integrated in all life domains, this study suggests a new terminology. We propose to move from insular ‘service ecosystems’ to ‘*Technology-mediated Life Ecosystems*’ that transcend the physical and digital business realms (e.g. event) and encompass all life domains of an end-user that are technologically-mediated and connected by AI.

### Practical implications

From a practical viewpoint, the study hopes to fill a timely and relevant gap in that it distilled a range of realistic scenarios of what the future of events could look like. Our findings shall support event planners in decision-making, implementation and experience design of AI in events. By offering plausible scenarios, this study helps businesses predict the use of AI, and thereby eliminates two of the most common errors, namely the under-prediction and over-prediction of change (Schoemaker 1995). In making a prognosis of the year 2026, the study has painted a picture of three distinct scenarios of how AI will have evolved to become an actor with agency in holistic life ecosystems that will shape the nature of human experiences - from highly positive personalised experiences to suspicious use, and to badly affected experiences. What is central to all scenarios is that AI, compared to previous technologies, offers a holistic system that connects customer-owned and business-owned technologies and facilitates personalised human experiences tailored to the last detail, thereby taking events to the next level. The core value proposition of AI thus lies in co-creating and personalising experiences, providing information and offering assistance, not only to attendees on a collective and large scale, but on the most granular level to the individual.

### Limitations and future research

Several limitations and suggestions for future research are defined. This study focused on events as a representative and highly technology-mediated business context. Data collection included all types of events (e.g. MICE, music festivals). The focus on one specific type of event may allow for a more nuanced understanding of AI specific to context, audience and situation. This study chose to consider various types of events to allow for wider scenarios of application and a broader contribution to the industry. AI is still in its infancy and largely theoretical, which posed challenges in imagination for the participants. It is recommended that further research builds on our futures-method study, and investigates real-life applications of AI to generate further solid contributions to understanding real life application. From a SD logic perspective, this study invites to address the following questions pertaining to the use of AI in service contexts: “What are the nuances and differences in human-to-non-human, compared to human actor-to-actor value co-creation? “How is agency negotiated and created in human-to-non-human value co-creation practices?” and “How can businesses extend their scope as stakeholders and technology providers in wider life ecosystems?” We hope to see these interdisciplinary questions taken forward in future service science, business, technology and marketing research.

## Appendix

**Table 3:** Full list of polar spectrum of 10 factors

Polar spectrum	Description
Factor 1 Suggestions/Assistance	
Projection A: Value co-creation (positive)	Attendees receive individual suggestions and assistance according to their needs. They can save time and make better decisions. AI improves the whole process and enhances the experience.
Projection B: Value co-destruction (negative)	Visitors get constant suggestions, which do not fit their needs. An overload of information leads people to become annoyed and feeling as though their experience and value is being co-destroyed. Participants miss some of their preferences, because the system gave wrong information.
Factor 2 Tracking of Customer Behavior	
Projection A: Subtle assistance	AI tracks every step and data of the customers in a discreet way and based on that, delivers good assistance to improve the experience.
Projection B: Totally controlled	Attendees feel controlled and they lack privacy. All their data is publicly accessible and not handled discreetly. Every attendee is transparent to others.
Factor 3 Type of Technology	
Projection A: Personal devices	AI appears in a form of wearable devices, smartphones, microchips, which are carried by the participants and the use of the system is obvious to everyone at the venue.
Projection B: Event technology	AI is used in the background and helps to improve processes, without the attendee recognising. Every technology used behind the scenes helps improve the customer experience.
Factor 4 Event Organisation	
Projection A: Value co-creation good development	Good development of the technology. Everything works properly and just some touchpoints are occupied with service staff.
Projection B: Value co-destruction bad development	The system does not work properly and needs constant interference of humans. It slows down whole processes, reduces revenue and attendees are annoyed.
Factor 5 Personalised Experience	
Projection A: Value co-creation positive experience	The whole customer journey is tailored to the attendees’ needs and helps to get the best out of the time. Products and prices are dynamic and personalised to the attendees’ needs. Attendees appreciate it and are open that AI helps them even to socialise and network with new people.
Projection B: Value co-destruction negative experience	The provided information is too general, and the attendees feel betrayed. Prices and products change constantly and obviously, so a sense of jealousy is created. Groups are torn apart and social entertainment is reduced. The overall experience is destroyed.
Factor 6 Crowd Management	
Projection A: Value co-creation good development	AI helps guide crowds according to capacity and security. The whole event can be simulated before the actual start and provides safety for all stakeholders. In case of emergencies, AI avoids mass panic or rather avoids emergencies through intelligent foresight.
Projection B: Value co-destruction bad development	AI is faulty and calculates incorrect scenarios. Visitor flows are misdirected and thus congestion, large crowds and dangerous situations arise. People cannot enjoy the event and therefore their experience is impaired.
Factor 7 Failure of System	
Projection A: Offline system	An overall AI system is developed, which is able to work offline. In case of network/energy problems, services still can be carried out.
Projection B: Total blackout	Network/energy problems create a total blackout. Systems do not work offline, and most services are offline. Access gates do not work anymore, and organisers do not have control over areas.
Factor 8 Orientation/Smart Guidance	
Projection A: Value co-creation positive guidance	AI provides individual guidance according to waiting lines and crowds, nevertheless, always has personal preferences in mind and leads based on the attendee’s interests.
Projection B: Value co-destruction negative guidance	AI acts in contradiction to what attendees want. It leads according to capacity and ignores individual preferences. Attendees miss chances and wants.
Factor 9 Security Concept/Surveillance	
Projection A: Value co-creation non-biased system	The system has an overview of the whole event and reduces emergencies. Event organsiers can adapt the system and therefore minimise risks. The aim is to improve the experience for the benefit of the customer.
Projection B: Value co-destruction biased system	The system does act in a sense of a higher power, which has specific bad intentions e.g. by collecting dataor by forcing attendees to action.
Factor 10 Marketing/Targeting	
Projection A: Suspicious	Attendees feel their data to be misused to increase sales. Every suggestion and call to action is only focused on the goal of increasing profits. They feel urged to buy something and do not trust the system.
Projection B: Trustworthy	AI proposes customised offers, depending on customers behaviours and preferences. Attendees feel understood and confirmed, and accept the technology unconditionally.
